# The role of part-time arrangements in the sustainability of midwifery continuity of care models in Australia: An integrative review

**DOI:** 10.18332/ejm/171359

**Published:** 2023-10-13

**Authors:** Olga Aleshin, Roslyn Donnellan-Fernandez

**Affiliations:** 1 The Royal Hospital for Women, Randwick, Australia; 2 School of Nursing and Midwifery, Griffith University, Nathan, Australia; 3 Transforming Maternity Care Collaborative, Griffith University, Meadowbrook, Australia

**Keywords:** midwifery, caseload, continuity, workforce, Australia, part-time

## Abstract

**INTRODUCTION:**

International maternity care experts have called for expanding midwiferyled continuity of care (MCoC) models. However, the number of models need augmentation as the number of women receiving this care is small. The majority of the midwifery workforce in Australian public health systems comprises women who work part-time. This aspect of the midwifery workforce demands careful consideration when attempting to change a maternity care system and sustain new models of care. Sparse research has been undertaken to explore whether part-time factors could play a role in the growth and sustainability of MCoC in Australia. This integrative review aims to analyze the role of parttime practice arrangements in the sustainability of MCoC models in Australia.

**METHODS:**

Following a systematic search of research databases (CINAHL, ScienceDirect, Cochrane Database of Systematic Reviews, and Proquest) and screening the literature with eligibility criteria including keywords related to midwifery continuity of care, workforce arrangements and full-time equivalent (FTE), eight Australian research articles were identified for evaluation. The articles were appraised for bias using the Mixed Methods Appraisal Tool (MMAT) and data were analyzed using an integrated convergent narrative synthesis method.

**RESULTS:**

The resulting themes from the synthesis suggest that part-time MCoC roles may support the sustainability of the MCoC workforce without reducing quality of care to women. In various studies, midwives reported that FTE (full-time equivalent) of 0.5 may not meet the job’s demands. However, this is likely influenced by local context and caseload size rather than the quantum of each midwife’s FTE. The quality of the studies is limited due to the small scale of the studies; however, the qualitative results give a depth of understanding to the strengths and challenges that part-time arrangements in MCoC add to the midwifery workforce.

**CONCLUSIONS:**

This review recommends that part-time arrangements in MCoC models in Australia be evaluated in conjunction with other routinely analyzed workforce data. Further considerations should be made by midwifery managers, leaders, stakeholders, and decision makers responsible for developing and supporting part-time job arrangements in caseload models of care in Australia.

## INTRODUCTION

### Rationale

Midwifery continuity of care (MCoC) is a relationship-centered model of care, considered increasingly as the gold standard for maternity care provision worldwide. Aside from clinical benefits, studies on maternal satisfaction with MCoC models have indicated aspects women value most from the relationships developed in MCoC: personalized care, empowerment, and trust^1,2^. There are various forms of midwifery-led MCoC models, provided by publicly funded services and midwives in private practice. In New Zealand, Lead Maternity Carer midwifery is nationally funded, yet midwives working in this role are autonomous practitioners providing access to home birth with visiting rights to hospitals^3^. This review will focus on the most common model provided in public hospitals in Australia – a caseload model of MCoC, which is often described as Midwifery Group Practices (MGP) or caseloading, where a midwife takes on a specified number of women per year (on average 35 women) per full-time equivalent (FTE)^4^. It is understood that MCoC models aim to provide women with a primary midwife and as much continuity with their primary midwife throughout their pregnancy, birth and postnatal continuum^5^. Given the international evidence supporting the benefits of MCoC models for women and infants^6,7^, national maternity care reform has called for increasing these models in Australia^8^. This has given rise to greater access to MCoC models, but access varies significantly by state, and the proportion of women receiving this model of care remains low^9,10^. In 2016, a national study found that 31% of the hospitals in Australia were providing MCoC with only 8% of women in Australia receiving MCoC^10^. In 2021, a separate study calculated that 18% of women in Queensland received MCoC care, demonstrating variation among states and territories^4^. However, in 2022 national data indicate the number of women receiving MCoC remains limited at 15%^9^.

Australian-based and international literature regarding the protective benefits of MCoC models on burnout and job retention is increasing^11,12^. The New Zealand arm of the WHELM study (Work, Health and Emotional Lives of Midwives) declared that midwives providing caseload MCoC were less burnt out and more satisfied with their work, even though they worked more hours on average than hospital employed midwives^13^. Research into the sustainability of MCoC models has illuminated some interesting factors contributing to midwifery retention in MCoC models. Maintaining similar caseload sizes and good relationships with partner midwives was identified as a leading contributor to sustaining the MCoC model in New Zealand, where Lead Maternity Carer midwives work autonomously and set their own caseload sizes within a self-employed structure^14^. The partnership developed with the women when providing caseload MCoC has also been described as a critical element that sustains midwives in MCoC models^15^.

Even with such promising evidence for MCoC models, the reality in Australia is that the number of MCoC models remains small, and most women in Australia continue to receive fragmented maternity care. Most employed midwives in Australia work part-time (on average 21.9 hours per week) and the vast majority are women (98.5%)^16,17^. Studies on the general midwifery workforce in Australia demonstrate that most of the part-time workforce is dissatisfied with a lack of flexible, family-friendly and relationship-centered job roles, motivating many midwives to leave the profession^18,19^. Attracting and retaining midwives within MCoC roles is challenging, with many non-MCoC midwives apprehensive about on-call requirements and work-life balance (WLB)^20^. Nevertheless, non-MCoC midwives often express an understanding that MCoC roles provide greater job-satisfaction, woman-centered care and autonomy, factors known to prevent burnout^20^. Moreover, increasing the availability of MCoC models so that midwives can fully utilize their education and training was a key recommendation of the federal government report into the midwifery workforce in 2019^9^.

Studies into the operationality of MCoC models in Australia have discovered that practice arrangements vary significantly among hospitals and states^21^. According to an Australian cross-sectional survey, of the 311 caseloading midwives working at most hospitals providing MCoC options, 276 worked at 1.0 FTE, meaning the majority of caseloading midwives (89%) were employed full-time^10^. Systematic reviews of midwifery-led MCoC consistently recommend investigating part-time options to sustain midwives in the profession^11,12^.

### Objective

This integrative review aims to analyze the current literature regarding the role of part-time workforce arrangements in the sustainability of midwifery Continuity of Care (MCoC) models in Australia. The development of the research question was guided using the SPIDER tool (Supplementary file Table 1). After reviewing various mixed-methods systematic review (MMSR) designs^22^ and utilizing the scoping review decision-making tool^23^, we chose to proceed with a modified five stage integrative review (IR) methodology based on Whittemore and Knafle^24^, which includes problem identification, literature search, data evaluation, data analysis and presentation. The IR allows for the inclusion of both empirical and theoretical literature, as well as mixed methods research (MMR), providing a richer extraction of information that could be used to guide future research, policies, and practice^25^.

## METHODS

Recent guidance for completing an integrative review affirms that IR methodology enables evaluation and synthesis of data from diverse sources to provide a comprehensive insight into what is known about a topic^26^. IR is best suited to the field of inquiry regarding the role of part-time workforce arrangements in the sustainability of MCoC models. This justification is based on rationale that IR methodology contributes to theory development combining theoretical and empirical evidence. Moreover, updated literature on the IR method identifies key strategies at each step of the review that increase rigor. The strategies, adopted in this review include two reviewers independently performing quality evaluation of the data, iterative critical analysis in the identification of themes and relationships in the data, and rigorous synthesis of the data based on critical consensus^25,26^.

### Search

Databases search included CINAHL, Cochrane Database of Systematic Reviews, Proquest, and Science Direct for eligible studies with geographical restriction to Australia. In addition, we sought input from midwifery networks and experts in the field of midwifery workforce issues in Australia.

*Eligibility criteria*

We included studies using qualitative and quantitative data collection and analysis methods. The main search string was: [midwifery OR midwives OR midwife] AND [continuity of care OR group practice OR caseload OR on-call], in combination with the keywords (keeping in mind the diversity in terminology within this topic area): part-time (part time), full-time (full time), job-sharing (job sharing), FTE (full-time equivalent), family-friendly, workforce, job satisfaction, maternal satisfaction, flexible, practice arrangement, retention, return-to-work, reduced hours, sustainability, burnout, and empowerment. There were no initial language or geographical restrictions. However, after consultation with the project supervisor, a decision was made to focus solely on the Australian context. As the initial National Australian Maternity Services Review was completed in 2009, with recommendations released in the National Maternity Services Plan 2010^8^, we limited the search period from January 2010 to August 2021. One article in-press during the initial search was subsequently published in March 2022 so the search period was updated to include January 2010 to March 2022. We limited our search to peer-reviewed articles. Studies were not excluded based on quality. Further studies were excluded if they involved: MCoC models that do not provide continuity of care in labor/birth (antenatal/postnatal continuity only), nursing workforce studies, studies that do not establish employment status, studies where only one sentence relevant to part-time work in MCoC was found in the discussion section (no prior mention), and opinion pieces. We want to highlight that there were many qualitative studies with valuable information on MCoC sustainability. However, these studies were excluded as they did not specify, or it was not easy to ascertain, the midwives’ individual FTE status.

*Search results*

**Figure 1:**
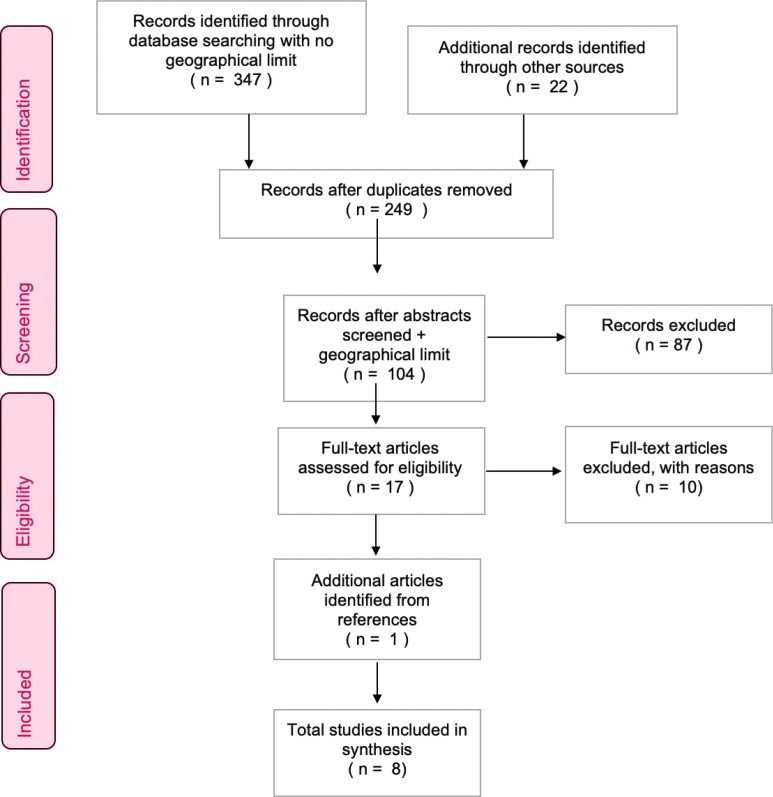
PRISMA flow chart

Results screened within the databases for relevance based on eligibility criteria were exported to Endnote. Study selection was documented using a PRISMA flow chart^27^ (Figure 1). We accessed 347 articles from databases and 22 from other sources. Duplicates were removed. After screening the abstracts, 104/249 articles remained. After the full-text screening, 17/87 articles remained. One additional relevant article was located from a reference citation^28^. Criteria were tightened at this stage to exclude articles concerning other countries (three international systematic reviews), articles based in New Zealand (two studies) and studies with insufficient information to draw conclusions (six articles). Eight studies were ultimately selected as relevant for this IR.

Given its validity in critically appraising a variety of health research designs, the updated version of the MMAT Tool 2018^29^ was selected to evaluate or appraise the mixed-method studies.

### Data extraction and analysis

There is no single method for analyzing and synthesizing the data obtained during the IR process^26^. A convergent integrated narrative approach to synthesize the mixed-methods studies was chosen^30^. Quantitative results were extracted using a narrative approach, by which results were written out ‘verbatim’ without interpretation. Qualitative results were summarized in similar narrative fashion. The summary of the descriptive findings was listed in a data table next to the relevant article. Two reviewers independently performed quality evaluation of the data. Any inconsistencies about the results of studies were clarified with the principal investigators of the studies. Descriptive findings were removed if they were not deemed pertinent to the concept of part-time positions affecting the sustainability of midwifery continuity of care models. At this stage, a rigorous, iterative process of comparison and contrast was adopted to reveal patterns and relationships in the data. Any outcome similarity between studies was color-coordinated to identify significant themes of commonality based on the frequency of discussion or geographical location. The themes were critically reviewed and agreed on during the writing process as listed in Table 1.

**Table 1:** Key findings

*Themes*	*Sub-themes*
Workload challenges	Ideal part-time FTE configurations and caseload size dependent on local context and workload Part-time hours are a challenge for leave arrangements
Recruitment	New graduates are a realistic way to increase recruitment into MCoC models Large number of newly graduated midwives interested in working part-time hours
Retention	Part-time hours may be protective of burnout, increasing job satisfaction and work-life balance Part-time hours mean greater time for selfcare and family
Acceptance from women	Part-time arrangements do not impact quality of care to women Women satisfied with care from part-time MCoC arrangements
Collaborative commitment to change	Developing MCoC models which include parttime arrangements is challenging Commitment from all stakeholders is essential for successful changes to be sustained

MCoC: midwifery continuity of care. FTE: full-time equivalent.

## RESULTS

### Study characteristics

The characteristics of each of the eight Australian studies are presented in Table 2, including geographical location (state/territory and rural/metropolitan). One pilot study was conducted at a tertiary hospital in Victoria and surveyed two part-time midwives (0.5 FTE) working in MCoC, eight full-time midwives working in MCoC, and 30 women who received care from the part-time midwives^31^. This study was small in scale and limitations were acknowledged. However, it is the first study to specifically research the influence of part-time employment in MCoC on women’s and midwives’ satisfaction. Two longitudinal studies were based in a metropolitan hospital in Melbourne, Victoria. They utilized the same data to evaluate different research questions related to establishing a new McoC model at the hospital^32,33^. These studies evaluated the work of caseload midwives, and compared the satisfaction and burnout of caseload versus non-caseload midwives. These studies were included because the authors accounted for FTE when analyzing their data and found no difference between part-time and full-time midwives. The qualitative portion of the study also gave insights into the part-time midwives’ perceptions of their workload and satisfaction. The two studies conducted in rural/remote settings in Queensland and South Australia were both longitudinal in design, and although having small sample sizes, had high response rates^34,35^. These studies both focused on implementing and upscaling MCoC models in their local areas. They included information on the FTE structure of their models and qualitative elements to give insight to staff experiences on their part-time arrangements. One study was a national cross-sectional survey of the perspectives of midwifery managers^28^. This study did not have respondents from the Australian Capital Territory. However, it had a high response rate overall, used consistent methodology and obtained information from 43 hospitals that provided MCoC models. The longitudinal mixed methods survey of newly graduated midwives in the Australian Capital Territory had a high response rate of 95/137. It gave a picture of what midwives’ desire and what would contribute to their job satisfaction^36^. This study gives critical evidence to factors contributing to the recruitment and retention of midwives entering the workforce. The qualitative study that interviewed 8 MGP midwives across Australia investigated the factors they perceive to contribute to the sustainability of MCoC models^37^. Midwives in this study came from different work environments and states and territories. Only one midwife interviewed identified as working part-time. At the time of the review this article was in-press and published in March 2022.

**Table 2:** Characteristics of included studies

*Authors Year Location*	*Study aims*	*Sample size*	*Design*	*Outcome measures*	*Findings related to part-time work arrangements*	*MMAT rating/reasoning*
Adelson et al.^35^ 2021 SA (rural) Australia	Evaluation of implementation, satisfaction and sustainability of the Midwifery Caseload Model of Care Pilot in rural South Australian setting	N=14 caseload midwives N=5 doctors N=6 core staff N=10 caseload midwives N=5 doctors N=9 core staff N=205 women (also interviewed stakeholders)	Mixed-methods studies including qualitative surveys and focus groups and quantitative data measurements	Maternal data, maternal interviews, staff interviews, stakeholder interviews, pre and post implementation and ongoing evaluations	Caseload and on-call challenging aspects Caseload of 38 per 1.0 FTE high, suggestions to review caseload and FTE requirements at different sites FTE began at 12.9 and increased by 2.8 over two-year period to accommodate for safety of caseload allocations Reduced caseloads for new graduate positions and managers Rural environments encompassing greater workload, longer distances travelled, administrative tasks Maternity leave and secondments addressed by providing core midwives opportunities for upskilling Collaborative relationships	Moderate quality evidence, high response rate – low bias for nonresponse rate Comprehensive evaluation with validated tools Small numbers representative of rural location
Dawson et al.^28^ 2018 Australia	Operationalizing caseload midwifery in the Australian public maternity system: Findings from a national cross-sectional survey of maternity managers	N=44 hospitals with caseload	Cross-sectional survey of maternity managers across Australia	Reveal the current practice arrangements, organizational barriers and facilitators and workforce requirements of caseload models in Australia	Broad range of FTE was used, ranging from 0.2 to full-time The majority of providers accepted midwives working 0.5–0.7 FTE arrangements	Moderate quality using descriptive quantitative analysis High response rate of 63% with valuable information on snapshot of MCoC models in Australia
Evans et al.^36^ 2020 NSW Australia	The future in their hands: Graduating student midwives’ plans, job satisfaction and the desire to work in midwifery continuity of care	N=95 graduating midwifery students	Longitudinal survey, mixed qualitative and quantitative	Uncover employment plans, early workforce choices, preferred models of care and the occupational motivators of graduating midwives in Australia	Majority of newly graduating midwives want to work part-time 60/94; of those wanting to work within midwifery, 47/91 want to work in CoC models Top three impacts on job satisfaction were less routine medical intervention, greater flexibility in working hours, greater number of midwives at work	Moderate quality: integrated both components of study Not generalizable to all midwifery population Those interviewed had exposure to MCoC models High response rate from Bachelor of Midwifery graduates Limitations discussed
Hewitt et al.^37^ 2022 Australia	Management and sustainability of midwifery group practice: Thematic and lexical analyses of midwife interviews	N=8 midwives working in MGP across Australia (NSW, WA, QLD, NT)	Qualitative	To explore optimal management of MGP in Australia and its influence on the sustainability of MGP	Working part-time allowed the midwives to cope with the work demands	Moderate quality qualitative methods – well described Triangulation achieved with lexical and thematic analysis Not generalizable to all Australian MCoC midwives – small sample
Newton et al.^33^ 2016 VIC (metropolitan) Australia	Understanding the ‘work’ of caseload midwives: A mixed-methods exploration of two caseload midwifery models in Victoria, Australia	N=21 66% caseload midwives worked part-time (at beginning of study)	Longitudinal quantitative and qualitative	Explore the views of caseload vs non-caseload midwives on new MCoC model	Caseload size impacted most on the personal life of caseload midwives, requiring ‘fluid navigation’ between home and work and good family support ‘Activity-based work’ beneficial to sustainability and managing WLB 5 midwives had primary school children MCoC midwives reporting more family time than shift work midwives	Moderate mixed-methods research using validated tools Did not acknowledge trend of change in part-time MCoC midwives (see findings in row below)
Newton et al.^32^ 2014 VIC (metropolitan) Australia	Comparing satisfaction and burnout between caseload and standard care midwives: Findings from two cross-sectional surveys conducted in Victoria,	N=150 (30 caseload midwives) 66% caseload midwives worked part-time (at beginning of study)	Longitudinal quantitative – questionnaires	Measure burnout/attitudes of midwives in new model of MCoC compared to standard care	No difference between full-time and part-time status after so can use this evidence to make conclusions about part-time Low burnout among caseload, higher satisfaction At baseline there were similar full-time and part-time with caseload and standard midwives Over the two years, the number of caseload midwives working part-time decreased from 65% to 36% (full- time increased from 35% to 64%) and the standard midwives’ part-time remained the same	Moderate quantitative research using validated tools Open ended questions coded to support findings Accounted for variations in population (caseload versus core midwives): burnout not impacted by the hours of work Attempted to address loss of participants and new participants Did not acknowledge trend of change in part-time MCoC midwives or explain meaning of ‘part-time hours excessive’
Styles et al.^34^ 2020 QLD (rural) Australia	Implementation and upscaling of midwifery continuity of care: The experience of midwives and obstetricians.	N=15 MW N=6 OBS After two years N=17 MW N=5 OBS	Qualitative Longitudinal	Perceptions of midwives and obstetrician of new MCoC model	Some midwives perceiving 0.5 FTE (20 h) not enough to provide MCoC Suggestions that greater FTE (some mentioning at least 0.8 FTE) for each midwife, and with even caseloads for each midwife would help the team address leave arrangements and allow the model to run more smoothly, i.e. workload would not be uneven, greater time for clerical tasks and meetings Collaborative relationships important	Moderate quality qualitative study using thematic analysis
Vasilevski et al.^31^ 2020 VIC (metropolitan) Australia	Satisfaction and care of women receiving part-time care and perceptions of midwives working part-time and full-time hours	N=30 women N=2 0.5 FTE MW N=8 1.0 FTE MW	Mixed-methods survey (online or phone) for women Online survey for midwives Quantitative data analyzed as descriptive statistics Qualitative data analyzed using ‘content analysis’	Self-reported surveys by women and staff regarding satisfaction of care and perception of part-time care	Approval by women with part-time model (28/30 satisfied, 19/30 found disadvantages Majority of midwives supporting part-time options Part-time midwives reporting less burnout and more satisfaction Recommendations for further considerations of MCoC models Suggestion for FTE to be at least 0.6 (rather than 0.5) to meet admin/meeting needs	Weak to moderate quality mixed methods study Self-reporting bias Discusses limitations

MMAT: mixed-methods appraisal tool. MMR: mixed-methods research. MGP: midwifery group practice. MCoC: continuity of care. MW: midwife. OBS: obstetrician. FTE: full-time equivalent. WLB: work-life balance. NSW: New South Wales. SA: South Australia. VIC: Victoria. QLD: Queensland. WA: West Australia. NT: Northern Territory.

## DISCUSSION

Four themes related to the sustainability of part-time work arrangements for MCoC models emerged from the review including: 1) *Workload challenges*, with sub-themes – Ideal part-time hours (FTE), caseload size, FTE distribution, and leave; 2) *Recruitment,* with sub-theme – Newly graduated midwives; 3) *Retention,* with sub-themes – Less burnout, greater satisfaction, and work-life balance; 4) *Acceptance from women,* with sub-theme – Part-time arrangements; and 5) *Collaborative commitment to change,* with sub-themes – Development of MCoC models, and commitment of stakeholders (Table 1).

### Workload challenges

*Ideal part-time hours (FTE)*

A critical analysis of the six studies revealed positive and negative factors related to part-time work arrangements in MCoC practices in Australia. In five of the studies ideal FTE fractions were discussed concerning caseload size and model-related workload. One study found that FTE varied greatly among practice arrangements, ranging from 0.2–1.0 FTE, but most services utilized 0.5–0.7 FTE arrangements^28^. In three of the studies, participants voiced perspectives that an FTE of 0.5 may not be sufficient to meet the demands of MCoC work including caseload size, clerical tasks, on-call and being available for regular meetings^31,34,35^. One rural midwife suggested a minimum of 0.8 FTE was ideal^34^, while a metropolitan-based study expressed 0.6 FTE as ideal^31^. This difference in perspectives of ideal FTE could be due to the differences in workloads for metropolitan versus rural MCoC services. In rural/remote studies, significant discussion surrounded factors that impact the caseload/workload of MCoC in rural areas including distances to travel, clerical/administrative obligations, increased education requirements, greater scope (social work roles), and limited resources. Midwives in these areas suggested an increase in total FTE as a way to address the increased workload, on-call challenges and leave arrangements in an area where staffing is a critical issue. This sentiment is reflected in the rural study which began with 12.9 FTE but added an additional 2.8 FTE by the second year to account for safety^35^.

*Caseload size and FTE distribution*

Caseload size per FTE was a significant workload issue that impacted midwives’ abilities to practice safely in MCoC models. One rural midwife suggested that calculating caseload using FTE needed to be better understood in implementing the model, which began with 30 women per FTE^35^. By the end of the second year of implementation, the caseload size was increased to 38 women per FTE. The second focus group discussion mentioned 38 women per FTE as being too high, given the complexities of rural workload. This midwife felt that a reduced caseload would improve the workload of midwives in rural settings, given the increased demands and limited resources experienced. Part-time working midwives in the metropolitan setting also expressed that caseload size impacted their ability to complete all tasks^31^. Similarly, midwives interviewed in the qualitative study found caseload size one of the most significant challenges in the MCoC model, with the part-time worker expressing that they could only cope with the workload because they worked part-time^37^.

Midwives also described the importance of having a team with equal FTE or workload distributed across the team^34^. They mentioned that having differing workloads would imbalance the team, creating more load on the midwife that works greater hours. This resonates with midwifery perspectives in a qualitative study in New Zealand in which midwives expressed having similar caseloads as necessary in sustaining the model^14^. With equal caseloads, the working midwife can handle their team mate’s load while the other team member has time off.

This review found that FTE structures and caseload size are significant, interrelated themes associated with part-time practice arrangements. These results reflect the findings of an integrative review that evaluated 22 studies (including thirteen from Australia and New Zealand) for elements related to the sustainability of caseload models of care^38^. A manageable caseload size has been identified as essential to sustainability and quality and safety concerns in MCoC studies^14^. The national caseload per FTE ranges between 36–40 women, and most hospitals adjust caseload based on workload, complexity of women, and service volume^28^. Given findings that excessive workload increases burnout in midwives providing MCoC^12^, this is particularly important for midwives who work part-time hours as they may require more time to complete administrative requirements of the role or time for travel, and other complexities involved in remote or rural caseloading work.

*Leave*

The studies in this review acknowledged leave as a significant challenge with practice arrangements in MCoC, which mirrors findings in the current literature^38^. Midwives interviewed found that planning annual leave was challenging^31,32^ as well as backfilling positions for unplanned extended leave periods^33^. The midwives who shared their caseload at 0.5 FTE each, expressed difficulty with the requirement by their hospital that they take their leave simultaneously^31^. Two midwives in the Victorian studies were on maternity leave during the follow-up period, highlighting the challenge of navigating a uniform workforce of primarily women^32,33^. To address the challenges of leave, core midwives were used to backfill positions, and give core midwives a chance to upskill^28,35^. Most maternity managers in the cross-sectional survey reported that leave arrangements were *ad hoc* – suggesting that this area of MCoC operationality is poorly managed. Studies have yet to identify if part-time MCoC options could support midwives’ returning to caseload work after maternity leave. Given that midwives returning from maternity leave are more likely to seek part-time arrangements, increasing part-time options and other creative strategies are essential to meet this need.

### Recruitment

*Newly graduated midwives*

Evidence shows that newly graduated midwives are interested in working in MCoC models. The longitudinal mixed-methods research study identified that most newly graduated midwifery students interviewed, desired to work in MCoC models and of the 95 participants, 65% desired to work part-time^36^. Hiring new graduates on a reduced caseload is a strategy suggested to recruit a greater number of newly graduated midwives into MCoC^35^. Only seven of the 43 hospitals in the cross-sectional survey currently recruited new graduates^28^. Increasing job placements for new graduates in MCoC with reduced caseloads, and in part-time capacities, may improve the recruitment of midwives into MCoC models and support the sustainability of these models.

### Retention

*Less burnout, greater satisfaction, work-life balance*

Burnout and job satisfaction are known factors that impact retention and sustainability in the midwifery workforce, including within MCoC models^12^. The findings of this review strengthen consideration that part-time hours may further protect against burnout, improve satisfaction and improve work-life-balance within this model. One midwife interviewed reported that she was happy to have moved to part-time arrangements because working full-time led her to burn-out, in a scenario that felt like she was always ‘catching her breath’ to survive^37^. High satisfaction and low burnout were identified in midwives providing MCoC compared to midwives providing standard care in the Victorian study^32^. The authors accounted for differences in populations and concluded that the hours worked did not impact the results, suggesting that caseload midwives working part-time had the same level of satisfaction as midwives working full-time, and that work-life balance was similar across participants. In the survey of midwives (n=14/18) providing MCoC in regional South Australia, there was an overwhelmingly positive response on empowerment and job satisfaction, and the second focus group also confirmed that the midwives were satisfied with their work^35^. All the midwives felt strongly that the MCoC was sustainable in the regional areas, and moderately satisfied with empowerment and work-life balance^35^. It may be valuable to research whether job satisfaction and WLB can be linked to reduced sick leave. In one study, 67% of respondents perceived that midwives working in MCoC models take less sick leave than non-continuity of care midwives^28^.

It was noted by midwives in the metropolitan Victorian study that working part-time allowed the midwives greater time to apply themselves to self-directed edification^31^. Midwives working part-time expressed having more time for their own interests outside of work, and were more likely to arrive at work well-rested and present. In addition to time for self, one study found that MCoC midwives reported having more family time than their shift-work counterparts^33^. If these results are interpreted as both part-time and full-time midwives in MCoC feeling this way, then it supports the notion that part-time arrangements benefit work-life balance in MCoC models. This aligns with the qualitative results from a large mixed-methods study which found that being a carer (to children or others) was the most significant obstacle to working in MCoC models^39^. It was suggested by the midwives surveyed in this study that flexible work arrangements and job autonomy were vital in navigating this work-life balance challenge. This supports the view that part-time arrangements increase work-life balance and are a valid solution to retention and sustainability of MCoC models.

In the longitudinal metropolitan studies, at baseline there were similar numbers of full-time and part-time midwives working in the MCoC model and midwives working in the standard hospital model^32,33^. Over the two years, however, the number of part-time caseload midwives decreased from 65% (n=13/20) to 36% (n=8/22) and full-time increased from 35% to 64%. The number of part-time midwives working in the hospital did not change. The authors attempted to address the loss and gain of participants but did not acknowledge this change in the FTE arrangement of the MCoC practice. Therefore, we cannot assess the reason behind this change: whether it was a sustainability issue or an organizational shift. Contact with the authors of this study for clarification of this phenomenon confirmed that the reduction in part-time positions reflected organizational decision making. Various studies reaffirm the need for good management and organization in order to protect work-life balance and sustain MCoC models^38^.

### Acceptance by women

Women’s perspectives were considered regarding quality care and satisfaction with receiving MCoC from a team of two part-time midwives (0.5 FTE)^31^. The vast majority of the women, (n=28/30) approved of the model of care. This study is the only one that specifically analyzed the impact of part-time midwifery care. A total of 205 women were surveyed in the South Australian study. Of the 52.6% of women who responded, 95% were optimistic about the care they received^35^. To support these results, focus groups at the beginning and end of the study asked midwives (n=14 and n=10), core nurses/midwives (n=6 and n=9) and doctors (n=5) whether they believed the women were satisfied with their care in the new model. There was overwhelming agreement from the staff that women were satisfied and that the model was woman-centered^35^. The results of this review suggest that part-time arrangements in MCoC models do not impact quality of care. Further research into the perspectives of women receiving care in MCoC models using differing FTE structures would benefit the knowledge base regarding this aspect of the sustainability of part-time positions in MCoC.

### Collaborative commitment to change

A significant theme from the focus group in the South Australian study was that the staff were committed to making the model work – signaling the understood value and improvement of this model as a service to the community^35^. One of the robust findings from this study was that all staff (not just the midwives) were committed to change and trying to find solutions to challenges. The Queensland study also described collaborative effort with aligned understanding of the benefits of the model as necessary^34^. Similarly, in a mixed-methods study describing the development of a MCoC program in rural New South Wales, a collaborative effort to implement a new MCoC model was attributed to the success of sustaining a MCoC model in an area that was threatened to lose the maternity services^40^. This suggests that the challenges of sustaining flexible part-time practice arrangements described, can only be met with a collaborative dedication to improvement. The cross-sectional survey of maternity managers found that 81% of hospitals with MCoC models utilized the Australian College of Midwives National Midwifery Guidelines for Consultation and Referral followed by health service specific guidelines (60%) and state guidelines (53%)^28^. It would have been helpful if this study explored in more detail the types of guidelines used by the health services to support the development of their practice arrangements.

### Limitations

The small number of studies with relevant evidence on part-time job arrangements is a limitation of this integrative review. In addition, many of the reports utilized self-reporting research techniques with a degree of bias that cannot be controlled. It must be appreciated that the differing contexts of practice including geographical locations where midwives in Australia work, likely impact the practice arrangements developed in each setting. However, it is promising to see pilot studies beginning to focus on identifying challenges and benefits of part-time arrangements. Research which specifies breakdown of FTE would be beneficial to understanding the impact of such arrangements on the sustainability of MCoC models in different settings.

### Implications

This review recommends further evaluation of MCoC models in Australia to explore the sustainability of existing part-time practice arrangements. The results of this review suggest that offering more part-time practice arrangements may provide a solution to sustain MCoC models in Australia. To remain sustainable, practice arrangements should be guided by area-specific needs, namely FTE distribution and caseload structures^41^. The literature synthesis in this review supports the premise that MCoC models may provide the female-dominant midwifery workforce in Australia with greater satisfaction and work-life balance. Moreover, the review highlights that these models of care are more attractive and sustainable if they incorporate flexible family-friendly employment, justifiable workloads, protected time off and supportive leadership^14,15^. Changing the organizational structure of existing MCoC practices and establishing new ones is multifaceted. This includes a consideration of non-hierarchical management structure, including leadership and workplace culture that values and embeds collective decision making and power sharing. Midwifery leaders who are committed to maternity care reform, and dedicated to advocating for midwifery staff should engage with the challenges outlined in this review to provide sustainable workforce solutions. Greater exploration of successful MCoC practice arrangements in diverse communities in Australia would assist policymakers, managers, and leaders to develop practice arrangements suitable to local settings^42^. It would befit policymakers to include strategies for sustainable part-time arrangements in guidelines and toolkits that can be accessed and shared nationwide.

MCoC models are accessible to only a small number of women in Australia even though there is strong evidence for expansion. Most of the midwifery workforce in Australian public health systems comprises part-time workers, but many midwifery continuity of care models only offer full-time employment arrangements. This review has provided evidence for the role of part-time arrangements in helping expand and sustain midwifery continuity of care models in Australia.

## CONCLUSIONS

Many questions remain regarding the role of part-time arrangements in the sustainability of MCoC models in Australia. The lack of studies suggests this is an area that needs more attention, given the diverse nature of the organizational structures of MCoC models around Australia. What this review adds to the knowledge of part-time employment arrangements for midwives working within these models is essential; that there may be significant benefits to workforce retention with more part-time MCoC options. Given the accumulating evidence that MCoC is the gold standard in quality care to women, and has greater satisfaction and less burnout for midwives, it is reasonable to surmise that this would be similar with part-time midwives. However, organizational factors have been identified as one of the biggest challenges to providing part-time arrangements for midwives including how managements address leave, caseload size, workload, and FTE structures. Further research into this area would help guide workforce policies and tools to give managers and midwifery leaders greater confidence in tackling practice arrangement challenges to meet Australian midwifery workforce needs.

## Data Availability

The data supporting this research can be found in the Supplementary file.
