# Supplementary material for: The role of part-time arrangements in the sustainability of midwifery continuity of care models in Australia: An integrative review
Source: Eur J Midwifery. 2023 Oct 13;7:27. doi: 10.18332/ejm/171359 (PMC10571291; doi:10.18332/ejm/171359)
Supplement: Supplementary file 1 [file EJM-7-27-s1.pdf]

Appendix

Table 1. SPIDER tool

(S) Sample	Midwives who work in midwifery-led continuity of care models (MCoC) (including caseload/midwifery group practice) which provide care throughout the pregnancy, birth and postnatal continuum.
(PI) Phenomenon of interest	The role of part-time workforce arrangements (including job-sharing, reduced hours, shared caseloads) in the sustainability of MCoC models in Australia.
(D) Design	Any research studies including qualitative, quantitative or mixed methods research (MMR). Limited by geographic location (Australia) and date range (2010-2022)
(E) Evaluation	Including but not limited to:<br>• Perspectives of midwives working in caseload models of care.<br>• Perspectives of student midwives exposed to caseload models of care.<br>• Perspectives of obstetricians working with caseload models.<br>• Perspectives of managers/other professionals working with caseload models.<br>• Quantitative information regarding employment arrangements that involve less than 1.0 FTE within caseload models of care.
(R) Research Type	Integrative review : synthesis of quantitative, qualitative and MMR using a convergent integrated approach and mixed method appraisal tools. A five stage design based on Whittemore & Knafl (2005) with a convergent integrated approach to data collection and evaluation.

© 2023 Aleshin O. and Donnellan-Fernandez R.
